# Temporal Monitoring and Predicting of the Abundance of Malaria Vectors Using Time Series Analysis of Remote Sensing Data through Google Earth Engine

**DOI:** 10.3390/s22051942

**Published:** 2022-03-02

**Authors:** Fahimeh Youssefi, Mohammad Javad Valadan Zoej, Ahmad Ali Hanafi-Bojd, Alireza Borhani Dariane, Mehdi Khaki, Alireza Safdarinezhad, Ebrahim Ghaderpour

**Affiliations:** 1 Department of Photogrammetry and Remote Sensing, K. N. Toosi University of Technology, Tehran 19967-15433, Iran; valadanzouj@kntu.ac.ir; 2 Department of Medical Entomology & Vector Control, School of Public Health, Tehran University of Medical Sciences, Tehran 6446-14155, Iran; aahanafibojd@tums.ac.ir; 3 Department of Civil Engineering, K. N. Toosi University of Technology, Tehran 19967-15433, Iran; borhani@kntu.ac.ir; 4 School of Engineering, University of Newcastle, Callaghan, NSW 2308, Australia; mehdi.khaki@newcastle.edu.au; 5 Department of Geodesy and Surveying Engineering, Tafresh University, Tafresh 79611-39518, Iran; safdarinezhad@tafreshu.ac.ir; 6 Department of Geomatics Engineering, University of Calgary, 2500 University Drive NW, Calgary, AB T2N 1N4, Canada; ebrahim.ghaderpour@ucalgary.ca

**Keywords:** malaria, remote sensing, climate, *Anopheles*, Google Earth Engine, hydro-climate time series, trend analysis

## Abstract

In many studies regarding the field of malaria, environmental factors have been acquired in single-time, multi-time or a short-time series using remote sensing and meteorological data. Selecting the best periods of the year to monitor the habitats of *Anopheles* larvae can be effective in better and faster control of malaria outbreaks. In this article, high-risk times for three regions in Iran, including Qaleh-Ganj, Sarbaz and Bashagard counties with a history of malaria prevalence were estimated. For this purpose, a series of environmental factors affecting the growth and survival of *Anopheles* were used over a seven-year period through the Google Earth Engine. The results of this study indicated two high-risk times for Qaleh-Ganj and Bashagard counties and three high-risk times for Sarbaz county over the course of a year observing an increase in the abundance of *Anopheles* mosquitoes. Further evaluation of the results against the entomological data available in previous studies showed that the high-risk times predicted in this study were consistent with an increase in the abundance of *Anopheles* mosquitoes in the study areas. The proposed method is extremely useful for temporal prediction of the increase in abundance of *Anopheles* mosquitoes in addition to the use of optimal data aimed at monitoring the exact location of *Anopheles* habitats.

## 1. Introduction

Malaria is an infectious disease transmitted by the *Anopheles* mosquito and claims millions of lives globally every year [1]. The pattern of malaria transmission varies markedly from region to region, depending on climate and biogeography [2]. Although malaria has been successfully eradicated in many parts of the world in recent decades, *Anopheles* mosquitoes have not become extinct. Furthermore, there is still the risk of malaria transmission in areas where *Anopheles* mosquitoes inhabit [3]. A recent study has shown that targeting the *Anopheles* larvae can be an effective tool in the fight against malaria [4]. The growth of *Anopheles* mosquitoes from eggs to larvae and finally to adult mosquitoes occurs in water bodies at a suitable temperature, thus the abundance of *Anopheles* mosquitoes is closely associated with the availability of precipitation, temperature and humidity [4,5,6,7,8].

Fortunately, malaria cases in Iran have dropped to zero in the last three years [9]. Therefore, according to the malaria elimination guidelines, the country represents a candidate for receiving the malaria elimination certificate. As the incidence of the disease decreases, it is expected that the budget allocated to the field-based operations, such as monitoring of the activity of larvae and adults of malaria vectors, which has previously been performed regularly in endemic areas of the disease, will be reduced. Therefore, access to the up-to-date data on *Anopheles* abundance and the accurate time of their temporal activity should be obtained through alternative means that are less expensive but more accurate. One of these ways is to study the environmental factors affecting the activity of malaria vector mosquitoes. Due to the fact that some environmental and climatic parameters affect the abundance of *Anopheles* mosquitoes, analysis and monitoring of climatic trends in the region will be effective in determining the accurate time for vector control interventions. The climatic trend of each region will be obtained by long-term monitoring of its climate data. Some studies on effective environmental parameters have shown that precipitation bears the greatest impact on the prevalence of malaria [10,11]. This phenomenon occurs due to the storage of water in pits and water bodies after rainfall. Precipitation is necessary to produce breeding sites for mosquitoes and for completion of its life cycle [12,13]. Moreover, temperature plays a vital role in the spreading of vector borne diseases. Temperatures between 13–35 °C are suitable for *Anopheles* mosquito breeding [14,15,16,17]. Land Surface Temperature (LST) marks one of the key parameters that can provide valuable information about the thermal characteristics of the ground, atmospheric effects of spectral radiation and bulk emissivity of the mixture of materials within the scene. Various satellites such as NOAA, Landsat, Terra, etc. have been designed for temperature studies. LST is positively associated with malaria incidence [12,18]. In addition to precipitation, moisture and LST, vegetation indices are also considered as one of the important environmental factors associated with the prevalence of malaria [3,11,19,20]. Temporal variation in Normalized Difference Vegetation Index (NDVI) reflects temporal agricultural and phenology changes and also tracks fluctuations in temperature and precipitation [21,22].

Vegetation indices, thus, provide an indirect measurement of the environmental pattern that affects the population of *Anopheles* mosquitoes. On the other hand, epidemiologic data of malaria cases are correlated with satellite based Vegetation Health (VH) indices [23,24,25]. The VH indices are represented by the Thermal Condition Index (TCI) and Vegetation Condition Index (VCI). The VCI and TCI estimate moisture and thermal conditions, respectively. Rahman et al. [25] discovered that the number of malaria cases was more sensitive to thermal (e.g., TCI) than moisture (e.g., VCI) conditions. Given that water areas provide the main habitat for the growth of *Anopheles* larvae, the water index such as Normalized Difference Water Index (NDWI) can also be considered as an effective parameter in the seasonal study of malaria prevalence [6,11,26]. Additionally, the index represents an indirect proxy for precipitation and humidity [27].

In previous research reviewed, not all effective parameters were analyzed simultaneously over a period of several years. In addition, field observations can be more accurate for monitoring effective factors, but the accessibility to this data is extremely limited in the long-term and over large areas. Certainly, in order to accurately predict the increase of the abundance of *Anopheles* mosquitoes, simultaneous examination of all parameters is important, and this analysis should be performed over a long period of time in the region [6,28]. In addition, the study of the correlations of environmental factors can be effective in their optimal selection to analyze and predict the abundance of *Anopheles*. Since most parameters affecting the increase of the abundance of *Anopheles* mosquitoes are climatic parameters, long-term analysis thus provides more accurate information about the climatic behavior of the region. If only one year is examined, the possibility of errors in the high-risk time prediction will occur. Some years experienced drought and the results of these years slightly differed from the typical climatic behavior of the region. Moreover, high-risk periods in a year can be detected using time series remote sensing data analysis. Therefore, by extracting the climatic and trends of environmental factors, high-risk periods within a year can be predicted. In recent years, Google Earth Engine (GEE) has rendered it possible to analyze time series remote sensing data easily and in the shortest time by providing fast and accessible processing space and easy access to free remote sensing data [29,30]. The aim of this study was to predict the high-risk time of increasing number of *Anopheles* mosquitoes and seasonal outbreak of malaria using time series of remote sensing data in three study areas of Qaleh-Ganj, Sarbaz and Bashagard counties in Iran. The remainder of the paper is organized as follows. Section 2 describes the study region, datasets, and methodology. Section 3 describes and illustrates the results. In Section 4, the results are discussed and reviewed.

## 2. Materials and Methods

**Figure 1:**
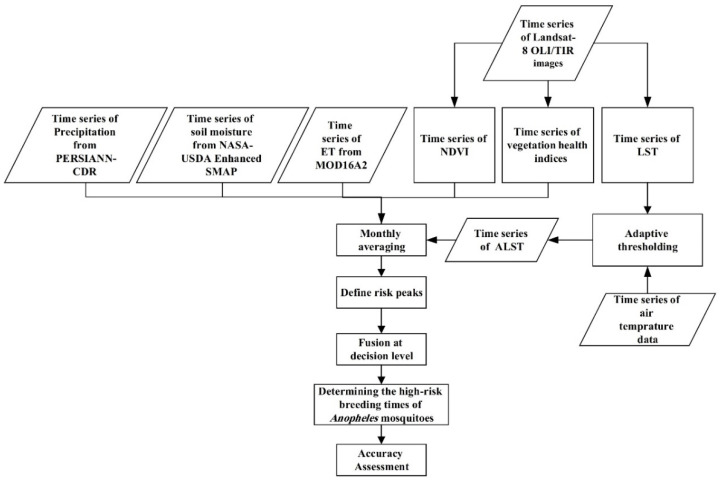
Flowchart of the steps of the proposed method.

In this research, high-risk times in a year among three study areas of Qaleh-Ganj, Sarbaz and Bashagard counties in Iran were studied by monitoring satellite-derived environmental factors over a period of seven years. According to this, time series of precipitation, LST, surface and subsurface soil moisture, NDVI and VH indices were analyzed simultaneously using GEE. The GEE contains the ability to analyze effective parameters on the abundance of *Anopheles* mosquitoes in the long-term by providing a strong processing space as well as easy access to time series remote sensing data. Each of these data contained its own temporal resolution, so as to equalize the temporal resolutions; the monthly average of each data was obtained. This would also be employed to check for high-risk months. In addition, the correlation between effective factors such as precipitation, ET, NDVI, Adaptive LST (ALST) and soil moisture were studied for optimal selection of parameters. Then, the risk peaks of each charts were calculated by examining the distribution of temporal data. Finally, by fusing the results of all effective factors over a period of seven years based on the majority voting decision, the high-risk months were identified in three study areas. To assess the proposed method, the results were evaluated against entomological data [31,32,33]. The flowchart of the proposed method is shown in Figure 1.

### 2.1. Study Region

**Figure 2:**
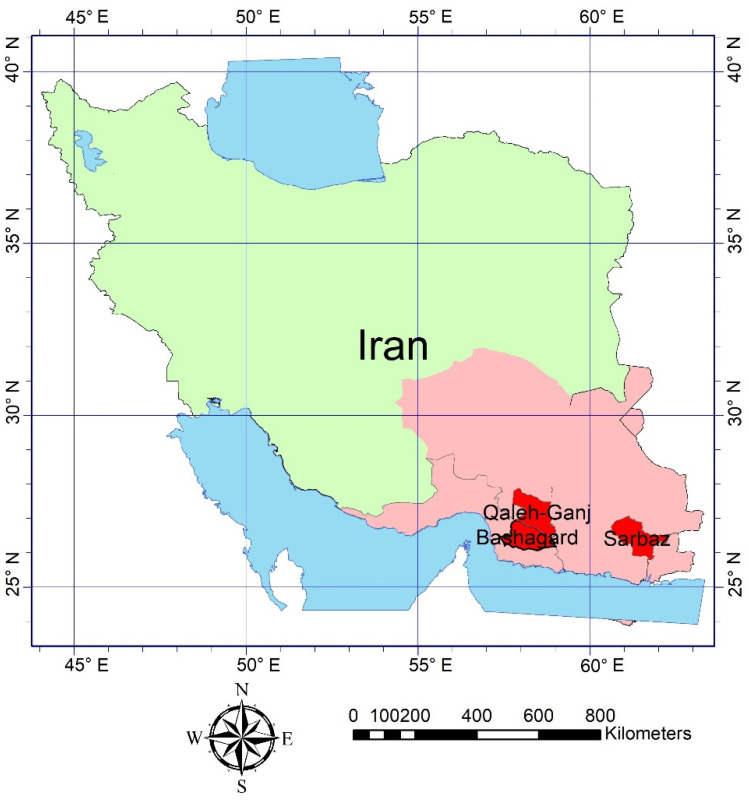
Three study areas including the counties of Qaleh-Ganj, Sarbaz and Bashagard (red) located in the three provinces of Kerman, Sistan and Baluchestan and Hormozgan, respectively, (pink), in Iran (green).

According to research conducted in Iran [33,34,35,36,37], in recent years, cases of malaria have been observed in the three counties of Qaleh-Ganj, Sarbaz and Bashagard. These three counties bear the potential to provide *Anopheles* mosquito larval habitats due to their subtropical climate and environmental conditions. The central point of Qaleh-Ganj county is located at 27°31′33.43″ Northing and 57°52′41.9″ Easting in the south of Kerman Province, the central point of Sarbaz county located at 26°37′58.28″ Northing and 61°15′30.01″ Easting in the south-east of Sistan and Baluchestan Province, and the central point of Bashagard county located at 26°27′29.08″ Northing and 57°54′7.39″ Easting in the east of Hormozgan Province (Figure 2). These study areas exhibit hot weather and monsoon rains in mid-summer. The study period in all three study areas ranged from 2014 to 2020.

### 2.2. Datasets and Pre-Processing

**Figure 3:**
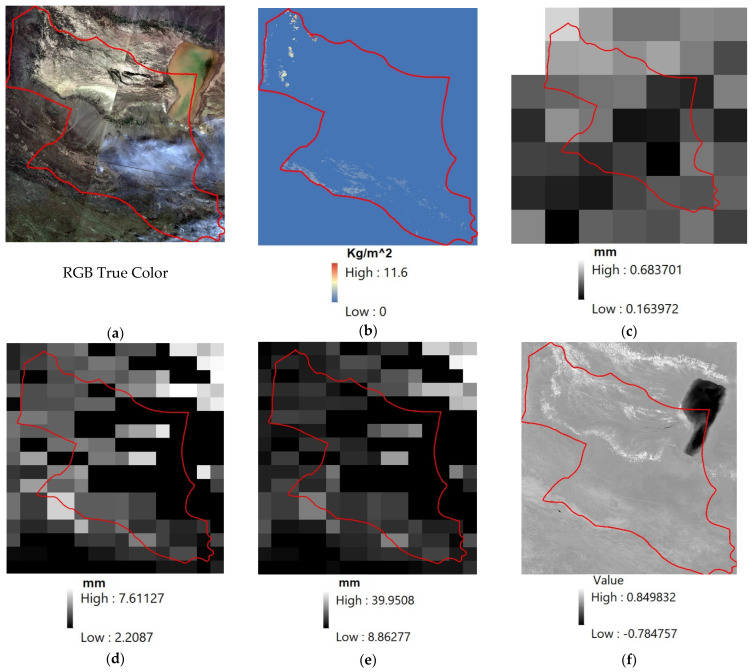

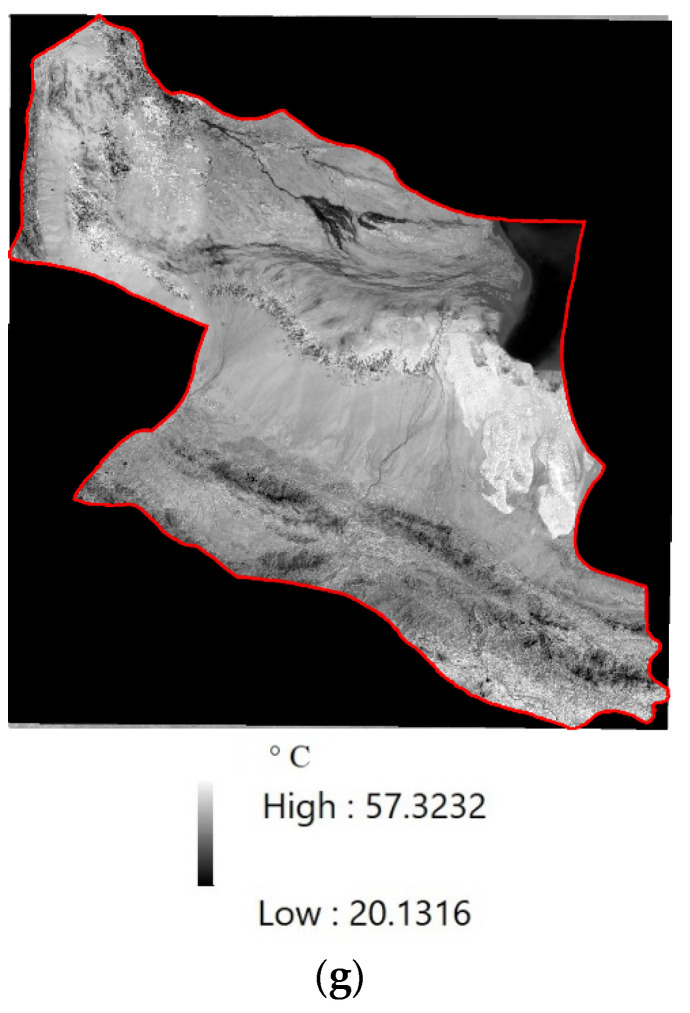
Sample data from Qaleh-Ganj County (Red Boundary) acquired in April 2020. (**a**) RGB true color; (**b**) ET; (**c**) Precipitation; (**d**) SSM; (**e**) SUSM; (**f**) NDVI; (**g**) LST.

Remote sensing data with medium and low spatial resolution were employed due to high temporal resolution, free usage, extended areas as well as relatively long study periods (Table 1). The data used include Landsat-8 OLI/TIRS satellite images to extract LST, vegetation and health vegetation indices, PERSIANN-CDR data to extract precipitation, MOD16A2 Evapotranspiration/Latent Heat Flux product to monitor ET and NASA-USD Enhanced SMAP data to monitor soil moisture (Figure 3). All these data were obtained as a time series for all three study areas of Sarbaz, Qaleh-Ganj and Bashagard counties from 2014 to 2020. The GEE, which is a cloud-based geospatial processing platform for large-scale environmental monitoring and analysis, was used to process the remote sensing data. This platform is a browser-based interactive development environment and a JavaScript programming interface provides access to a wide range of satellite products. Computations in this environment are performed through parallel processing in Google Cloud [30].

In addition, due to the importance of temperature conditions in the growth of *Anopheles* larvae, a series of meteorological data from the beginning of 2014 to the end of 2017 were used to better adapt temperature with LST factor. These data were prepared by the Iran Meteorological Organization (IMO). The meteorological data include the minimum and the maximum temperature per day from the synoptic stations of the study areas.

#### 2.2.1. Adaptive Land Surface Temperature (ALST)

*Anopheles* mosquitoes encompass different species and each of them grows and survives within a certain temperature range. In general, most of the *Anopheles* larvae species developed into adults at temperatures ranging from 13 to 35 °C [14,15,16,17,38]. In this research, LST time series from Landsat 8 OLI/TIR images were used to monitor land surface temperature changes. In order to calculate the surface temperature, first, it was necessary to apply pre-processing on the images including applying cloud and shadow masks in addition to atmospheric and radiometric corrections. The NDVI was applied (Equation (1)) to determine the emission capacity of the land surface.

(1) $$\mathrm{NDVI}=\frac{\mathrm{NIR}-\mathrm{Red}}{\mathrm{NIR}+\mathrm{Red}}$$

where NIR and Red are equal to the near infrared and the red bands, respectively. After calculating the NDVI index, based on the obtained values, Land Surface Emissivity (LSE) was determined according to Table 2 [39].

The BT is the temperature corresponding to the radiance received from the surface of a phenomenon or object by the sensor, which is obtained by the inverse of the Planck relation (Equation (2)).

(2) $$\mathrm{BT}=\frac{k_{2}}{\ln\frac{k_{1}}{L_{\lambda }}+1}$$

where L_λ_ is equal to the spectral radiation, λ is the central wavelength of each band, and k_1_ and k_2_ are equal to the calibration coefficients of the sensor brightness temperature.

Then, the surface temperature is calculated through Equation (3) [39].

(3) $$\mathrm{LST}=\frac{\mathrm{BT}}{1+\left(\lambda \;\frac{\mathrm{BT}}{\rho }\right)\ln\left(\varepsilon \right)}$$

where λ is the band wavelength and ε is equal to the LSE (Table 1). ρ can also be calculated from Equation (4).

(4) $$\rho =\frac{\mathrm{hc}}{s}=1.438\times 10^{-2}\;\mathrm{mk}$$

where h is the Planck constant (6.626 × 10^−^^34^ J.s), c is the speed of light (1.38 × 10^−^^23^ J/K) and s is the Boltzmann constant (2.998 × 10^−^^8^ m/s). Finally, in order to convert the LST unit from Kelvin to Celsius, the value of 273.15 is reduced.

In describing the correlation between temperature and the abundance of *Anopheles*, the mean value of meteorological data for the whole study period was chosen to correct the LST results. For this purpose, the average monthly air temperature was calculated from the average minimum and maximum temperature per day, and then the average LST per month was matched with the corresponding average air temperature. This analysis was performed for four consecutive years from 2014 to 2017. Based on this analysis, the monthly median value of difference for four consecutive years was extracted as the monthly adaptive LST threshold. It should be mentioned that considering the median value could reduce the effective of noisy data in thresholding process.

#### 2.2.2. Precipitation

One of the methods for estimating precipitation is the use of Precipitation Estimation from Remotely Sensed Information using PERSIANN-CDR data. PERSIANN-CDR provides daily rainfall estimates for the latitude band 60° S–60° N. PERSIANN-CDR is aimed at addressing the need for a consistent, long-term, high-resolution (0.25 degree), and global precipitation dataset for studying the changes and trends in daily precipitation, especially extreme precipitation events, due to climate change and natural variability [40]. In this study, in order to monitor the precipitation trends of the study areas, PERSIANN-CDR data from 2014 to 2020 were averaged on a monthly basis using GEE.

#### 2.2.3. Soil Moisture

NASA-USDA Enhanced SMAP global soil moisture data provides soil moisture information across the globe at 10-km spatial resolution. This dataset includes surface and subsurface soil moisture and was also created by integrating SMAP surface soil moisture satellite-derived observations into a modified two-layer Palmer model using a one-dimensional (1D) ensemble Kalman filter (EnKF) data assimilation approach. The integration of SMAP soil moisture observations helps improve the model-based soil moisture prediction, especially in areas of the world that lack good quality precipitation data [41]. In order to study changes in soil moisture over the period of seven years, the monthly average of surface and subsurface soil moisture were obtained in GEE over the study areas.

#### 2.2.4. Normalized Difference Vegetation Index (NDVI)

One of the most widely employed and simplest vegetation indices, used to monitor vegetation changes, is the NDVI (Equation (1)). As outlined in the literature, changes in this index are directly related to the prevalence of malaria. In this study, the NDVI was extracted as a monthly average, from 2014 to 2020, using red and near infrared bands of the Landsat 8 OLI. This implementation was performed in the GEE for all study areas.

#### 2.2.5. Vegetation Health (VH)

The most widely used indicators of vegetation health and drought are VCI and TCI. If TCI increases and VCI decreases during the time, this means that drought has occurred in that area [42]. Apart from the fact that these two indicators are employed in vegetation health and drought, they monitor vegetation and temperature in their equations in the long-term; thus they can also be used as an indicator of the ideal growth conditions of *Anopheles* mosquitoes. Decreasing the values of VCI over a long period of time indicates a decrease in vegetation moisture. This index compares the current NDVI to the range of values observed over a period of time. The VCI index is defined as follows (Equation (5)) [43,44].

(5) $$\mathrm{VCI}=\frac{{\mathrm{NDVI}}_{i}-{\;\mathrm{NDVI}}_{\min}}{{\mathrm{NDVI}}_{\max}-{\;\mathrm{NDVI}}_{\min}}$$

where, ${\mathrm{NDVI}}_{\max}$ and ${\mathrm{NDVI}}_{\min}$ are equivalent to the maximum and minimum NDVI over the study period, respectively, and i represents the current month.

The TCI is used to monitor vegetation drought changes over a long period of time. This index is based on the relationship between actual (${\mathrm{LST}}_{i}$) and potential condition temperature (${\mathrm{LST}}_{\min}$) and vegetation stress (${\mathrm{LST}}_{\max}$) (Equation (6)) [43,44].

(6) $$\mathrm{TCI}=\frac{{\mathrm{LST}}_{\max}-{\;\mathrm{LST}}_{i}}{{\mathrm{LST}}_{\max}-{\;\mathrm{LST}}_{\min}}$$

where, ${\mathrm{LST}}_{\min}$ and ${\mathrm{LST}}_{\max}$ are equivalent to the minimum and maximum LST over the study period, respectively, and i represents the current month. The increasing trend of TCI in the long period of time demonstrates vegetation drought in the region [42]. In this research, the TCI and the VCI had been implemented as a time series from 2014 to 2020 for all study areas. Accordingly, both annual and 7-year components of maximum and minimum of NDVI and LST were used to calculate VCI and TCI. Since both VCI and TCI were calculated from NDVI and LST, respectively; the correlation of these indices with their related factors would be determined to reduce the feature space.

#### 2.2.6. Evapotranspiration (ET)

The MOD16A2 Evapotranspiration/Latent Heat Flux product is an 8-day composite product produced at 500-m pixel resolution by MODIS sensor. The algorithm applied for the MOD16 data product collection is based on the logic of the Penman–Monteith equation, which includes inputs of daily meteorological reanalysis data along with MODIS remotely sensed data products such as vegetation property dynamics, albedo, and land cover [45]. The pixel values for the ET represent the sum of all eight days within the composite period. Since the ET is related to soil moisture, water content of vegetation, temperature and precipitation, this component could also be one of the factors considered in the malaria prevalence. Therefore, in this study, the monthly average of ET changes from the MOD16A2 product in the seven-year period were applied using the GEE platform.

### 2.3. Determining the High-Risk Breeding Time of Anopheles Mosquitos

After calculating the monthly average of all seven parameters of NDVI, LST, ET, soil moisture, TCI, VCI and precipitation, their seven-year average per month was also determined. In this way, over a period of seven years, the climatic trend of the region affecting the abundance of *Anopheles* mosquitoes was estimated. In order to define the risk peaks, the average line of box plot for each environmental factor was used. Sudden changes in the average line between two consecutive months were defined as the start and the end of a risk peak. In this way, we can obtain a better observation at the distribution of data over time series, especially regarding the months that exhibited variable behaviors in consecutive years (with high standard deviation). Then, a fusion of the results at the decision level was used to identify high-risk months in which the necessary conditions were provided for the growth and development of *Anopheles* mosquitoes. In this method, the majority voting condition was used in the final decision. Thus, if out of every six parameters, four or more parameters occurred simultaneously in each month (in its peak interval), that month would be selected as the high-risk month. It should be noted that temperature plays a definitive role in this process and the appropriate temperature for *Anopheles* mosquitos’ growth must be considered in the final decision.

## 3. Results

The time series of LST, precipitation, soil moisture, NDVI, vegetation health indices and ET were implemented in GEE.

### 3.1. Land Surface Temprature (LST)

**Figure 4:**
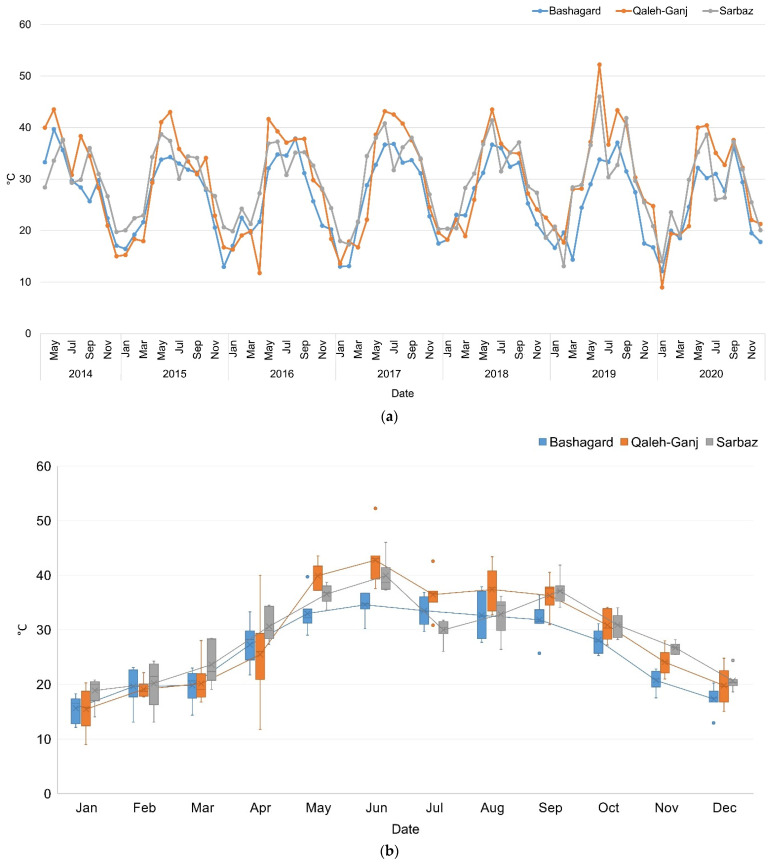
(**a**) Adaptive LST time series; (**b**) Box plot of adaptive LST time series of Qaleh-Ganj, Sarbaz and Bashagard counties, Iran, 2014–2020.

LST is directly related to temperature; therefore, to analyze the time series of temperature, the monthly average LST from Landsat-8 OLI/TIR satellite images were extracted. Moreover, in order to better adapt the LST to the air temperature to further accurately determine the high-risk times within a year, the 4-year time series of meteorological data from synoptic stations in all three study areas were analyzed. The median values of the differences between the air temperature and LST in the same months from 2014 to 2017 were calculated and these values were applied as adaptive thresholds on the initial time series of LST (Table 3). The ALST time series for the three study areas of Qaleh-Ganj, Sarbaz and Bashagard counties are shown in Figure 4.

Finally, it was possible to use the air temperature thresholds of growth and survival of *Anopheles* larvae on ALST time series data and predict high-risk times in each study area. Therefore, the suitable ALST for the growth and survival of *Anopheles* mosquitoes approximately ranged from 15 to 35 °C.

### 3.2. Normalized Difference Vegetation Index (NDVI)

**Figure 5:**
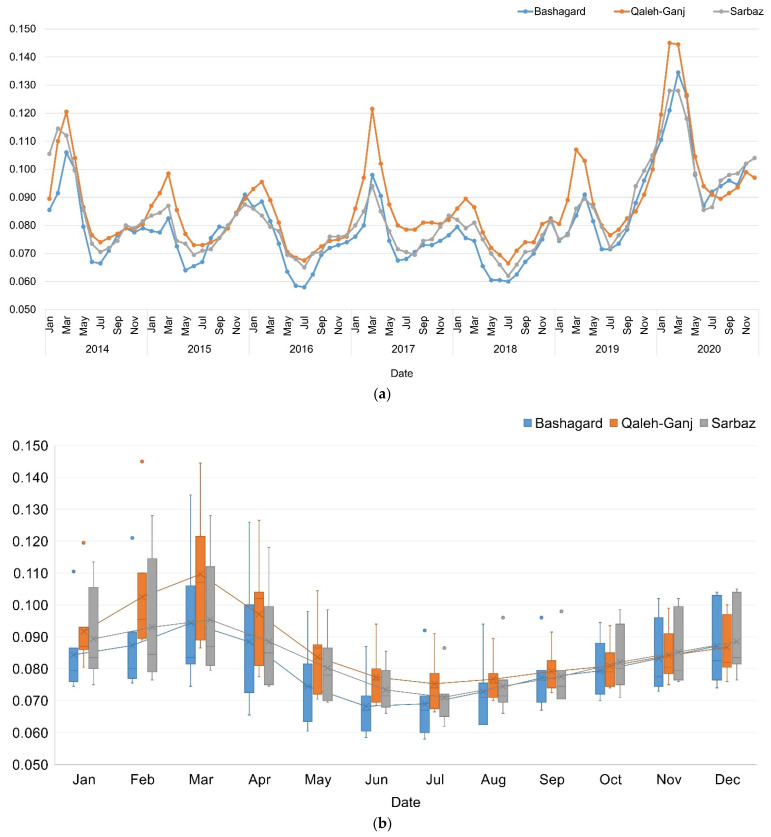
(**a**) NDVI time series; (**b**) Box plot of adaptive NDVI time series of Qaleh-Ganj, Sarbaz and Bashagard counties, Iran, 2014–2020.

Vegetation changes in study areas were analyzed using NDVI time series extracted from red and near infrared bands of Landsat-8 OLI images. In this process, surface reflectance images with cloud coverage of less than 5% were applied. As can be seen from Figure 5, the NDVI values for all three study areas were extremely low and close to zero. These results indicate lack of vegetation in these areas.

Based on the mean line in Figure 5b, there was a peak for all three study areas from August to March each year. The decrease in NDVI could be due to the high temperature and drought in the summer months and its increase in winter might be due to the decrease in temperature and drought, as well as the cultivation of winter crops.

### 3.3. Precipitation

**Figure 6:**
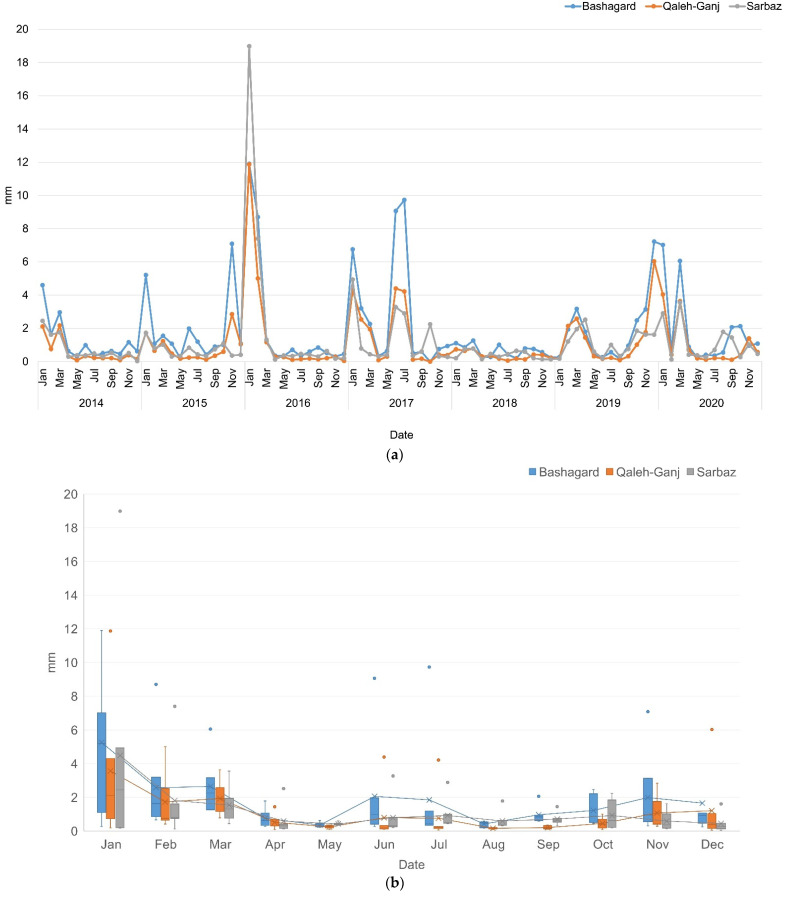
(**a**) Precipitation time series; (**b**) Box plot of precipitation time series of Qaleh-Ganj, Sarbaz and Bashagard counties, Iran, 2014–2020.

Another important parameter in increasing the abundance of *Anopheles* was precipitation. The average daily precipitation chart within a month was prepared employing the daily precipitation data of PERSIANN-CDR in GEE from 2014 to 2020. The horizontal axis of the chart represents the months of a year and, its vertical axis displays the average of precipitation in millimeters for each month. The outliers in the box plot indicate the rainy years within the study areas, which extended beyond the average rainfall in the long-term (Figure 6b). 

### 3.4. Soil Moisture

**Figure 7:**
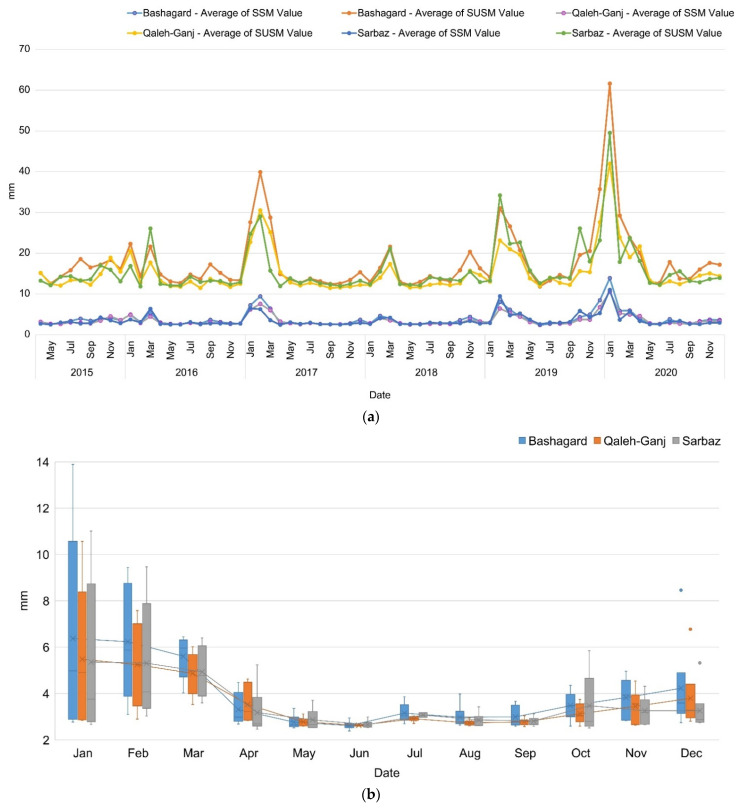
(**a**) Soil moisture time series; (**b**) Box plot of soil moisture time series of Qaleh-Ganj, Sarbaz and Bashagard counties, Iran, 2015–2020.

Soil moisture represents another effective environmental factor. Relative moisture affects the lifespan of *Anopheles* mosquitoes. Soil moisture of the study areas is shown in Figure 7. In this figure, soil moisture was generated in two forms of Surface Soil Moisture (SSM) and Subsurface Soil Moisture (SUSM) using the monthly averaging of NASA-USDA Enhanced SMAP time series data. Based on these results (Figure 6b), all three study areas exhibited the highest levels of SSM and SUSM from January to the end of April and October to the end of December. A short peak was seen in July, which may be related to summer rainfall.

### 3.5. EvapoTranspiration (ET)

**Figure 8:**
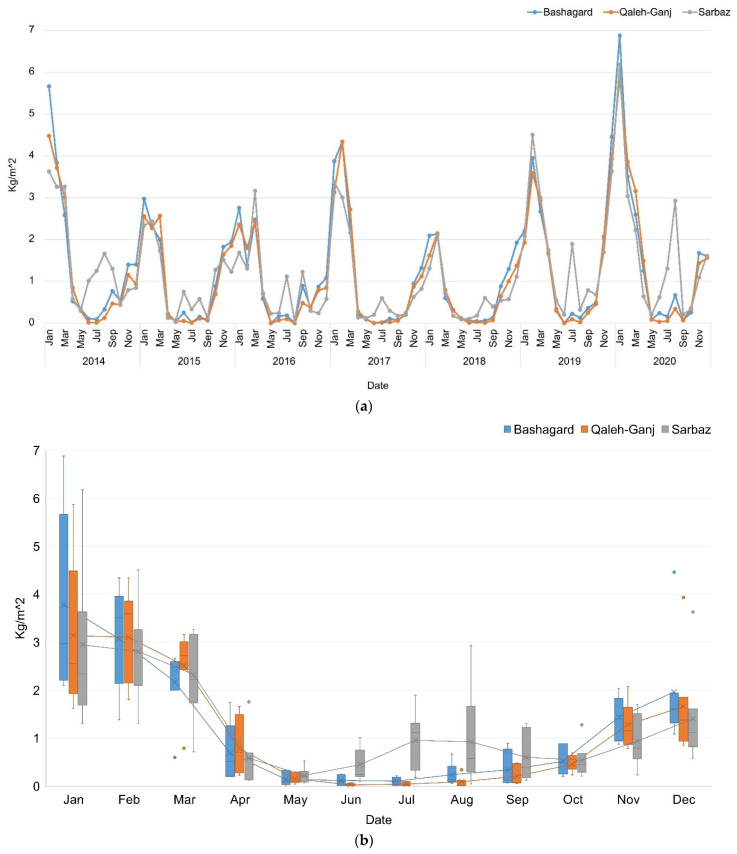
(**a**) ET time series; (**b**) Box plot of ET time series of Qaleh-Ganj, Sarbaz and Bashagard counties, Iran, 2014–2020.

The ET represents the next factor, theoretically related to the NDVI, LST and SSM. Therefore, it was necessary to examine the correlation between these factors to reduce the feature space. The risk peaks of ET for Bashagard and Qaleh-Ganj were in January to May and September to December. In addition to the mentioned months, an extra peak of ET was observed in July and August in Sarbaz (Figure 8).

### 3.6. Vegetation Health (VH)

The last factor monitored in relation to the abundance of *Anopheles* mosquitoes was vegetation health indices. Monitoring the time series of these indices over a long-time period could also reveal the drought trend in the study areas. Both annual and 7-year components of NDVI and LST were used to calculate VCI and TCI. The correlations between vegetation health indices and their related parameters including NDVI and LST were calculated in Table 4 and Table 5. According to the results, the correlation between LST and TCI measured extremely high in both annual and 7-year components cases, but for VCI, there was a medium correlation for annual component case. Therefore, it might be useful to retain this factor as a result of temporal prediction; and also, due to the high correlation between TCI and LST, this factor was excluded from the results. The annual components of the time series of LST and TCI are out-of-phase while, the annual components of NDVI and VCI are in-phase.

**Figure 9:**
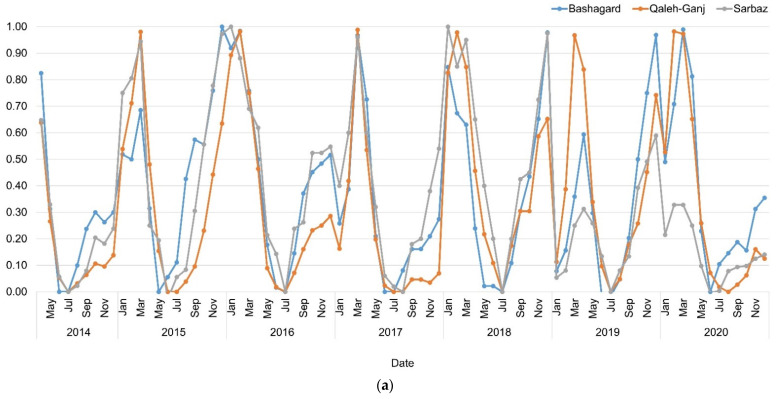

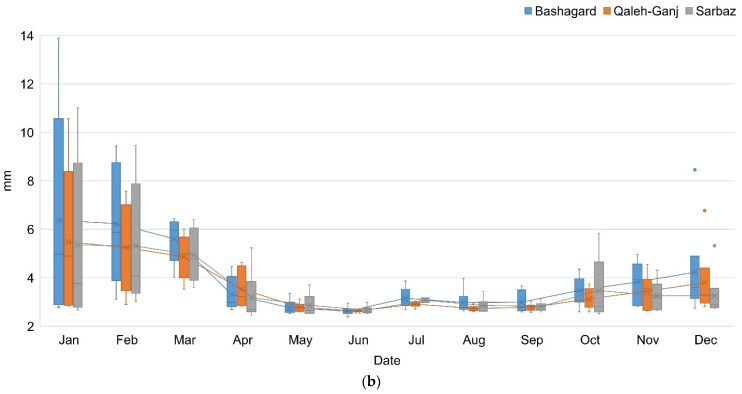
(**a**) VCI time series; (**b**) Box plot of VCI time series of Qaleh-Ganj, Sarbaz and Bashagard counties, Iran, 2014–2020.

Based on the mean line in Figure 9b, there was a peak for all three study areas from August to April each year.

### 3.7. Data Fusion and Determining the High-Risk Time

**Figure 10:**
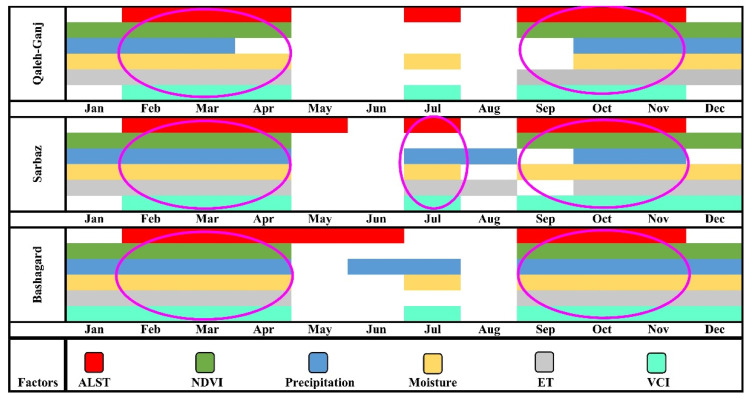
Temporal prediction based on the averaging of effective parameters in increasing the *Anopheles* abundance over a period of seven years. Pink ellipses indicate high-risk times within a year for study areas.

Finally, all the above results for the parameters of ALST, NDVI, precipitation, soil moisture, ET and VCI are shown in Figure 10. Temperature plays an essential role in the growth of *Anopheles* larvae representing the main factor. Therefore, ALST was involved as an important feature in fusing the results at the decision level, but other parameters played a less important role in the final results. On the other hand, some factors were correlated with each other and provided common information. The magenta ellipses in Figure 10 illustrate the simultaneous occurrence of the peak of most factors. These represented high-risk times for the growth of *Anopheles* mosquitoes, and then, if there were cases of malaria in the area, it will correlate to the spread of this disease. Simultaneous temperature and precipitation provide suitable conditions for the growth of *Anopheles* larvae, but the presence of a high amount of ALST (more than 35 °C) from May to September in the study areas, provides an important factor in which the absence of cool shelter renders it impossible for the growth and survival of *Anopheles* mosquitoes.

Table 6, Table 7 and Table 8 show that ALST, ET and TCI had exhibited a high correlation between each other, and the rest of the factors in all three study areas had correlation coefficients greater than −0.7 and less than 0.7. The types of analysis of LST and ET were based on a thresholding process and risk peak determination, respectively. Therefore, these two factors may bear different results in the final decision.

## 4. Discussion

In this article, high-risk periods for three counties in Iran were monitored and predicted using remote sensing data in a seven-year time series through GEE. The three parameters of temperature, precipitation and vegetation as effective parameters in previous research were examined separately or in combination with remote sensing satellite observations and meteorological field observations during short periods. Given that the aim of this research was the prediction of high-risk periods that would increase the abundance of *Anopheles*, it was necessary to monitor the climatic conditions of the study areas over a long period of time. Based on this, seven parameters of ALST, NDVI, precipitation, soil moisture, ET and VH indices were monitored over a seven-year period by processing time series of remote sensing data in GEE. Due to GEE’s valuable capabilities regarding easy access and fast processing of remote sensing time series data, it was possible to monitor a variety of environmental parameters that directly or indirectly provide the suitable conditions for *Anopheles* mosquito growth and survival; and, consequently, malaria outbreaks in susceptible areas.

According to the results obtained in Figure 10, two peaks in a year for Bashagard and Qaleh-Ganj counties were identified as high-risk times. The first peak stemmed from the middle of winter to the middle of spring, and the second peak was from late summer to mid-autumn. Apart from the two high-risk times of the year similar to the high-risk times in Bashagard and Qaleh-Ganj counties, Sarbaz exhibited another high-risk time in the middle of summer due to summer rains.

The co-occurrence of appropriate values of ALST, precipitation, NDVI, soil moisture and VCI would render this period of time the most high-risk time within a year concerning the study areas. During the summer months of June and July, precipitation, LST and high VCI were observed. If the ALST reaches above 35 °C on average in summer, temperatures are not suitable for the growth and survival of *Anopheles*. Furthermore, if large pits and depressions are formed, e.g., through monsoon rains in the study areas, they will exhibit low stability due to the high dryness of the regions. Pits and depressions will grow dry due to surface evaporation and high permeability of dry soil, and, consequently, *Anopheles* larvae will lack sufficient time to grow in an aquatic environment. These conditions will be effective for the growth of *Anopheles* larvae only when the vegetation in the area is high and gardens and farmlands are available. Since these areas can provide a cool shelter for the survival of *Anopheles*, and if water accumulates at the base of trees and shrubs, they can create a larval habitat. Therefore, over time, the larvae develop into adult mosquitoes (higher temperatures within the appropriate temperature range accelerate this process) and cause the spread of malaria in the region.

In order to evaluate the accuracy of the estimated high-risk times in a year, field data of the abundance of *Anopheles* mosquitoes were required. Due to the lack of access to appropriate entomological data in the regions, the previous three studies [31,32,33] conducted in the study areas were employed for final evaluation. The years studied in these three research works represented neither rainy nor drought years and were consistent with the average climatic conditions over a seven-year period.

According to a study conducted by Edalat et al. [33] in Qaleh-Ganj County, the abundance of *Anopheles* mosquitoes began to increase in February and had been increasing until May, after that, the abundance rate became negative. Again, from September to November, the abundance of *Anopheles* mosquitoes increased and then decreased rapidly in December. These statistics were obtained for five different species of *Anopheles* mosquitoes, including *An. stephensi*, *An. culicifacies* s.l., *An. superpictus* s.l., *An. dthali* and *An. fluviatilis* s.l. All five species exhibited similar behaviors in increasing and decreasing abundance.

The next study was conducted in Sarbaz County on four different species of *Anopheles* mosquitoes, including *An. stephensi*, *An. culicifacies* s.l., *An. superpictus s.l*., *An. dthali* and *An. fluviatilis* s.l., from 2016 to 2017 [30]. Apart from *An. culicifacies*, three other species started to increase in abundance in early February up to April and then decreased until June. The abundance rate had increased again since September and then decreased since the beginning of November. In *An. culicifacies* species in both mentioned periods, it had increased one month earlier and decreased one month later. By comparing the results of the proposed method (Figure 9) with entomological data for Qaleh-Ganj and Bashagard counties, the accuracy of the predicted high-risk times was confirmed.

Nejati et al. [32] performed abundant counting on only one type of *Anopheles* species called *An. subpictus* in Sarbaz County. According to this research, three major peaks were observed in 2015. The first peak began in February, peaked in March and declined in April. The second peak was observed in July and August, and finally, at the beginning of September, the third peak started and reached its maximum in early December.

By comparing the research results with the entomological data of Sarbaz County, three time periods from February to May, July and also from October to November were confirmed.

Although the annual average of effective factors was separate from the monthly study of them, it was useful to display the impact of each factors on the prevalence of malaria. Unlike other parameters, temperature was associated with a delay in the abundance of *Anopheles*. Temperature represents one of the most important factors in the growth of *Anopheles* larvae, but in the results of quantitative description exhibited a low correlation. This is due to an increase in temperature at the end of winter and the provision of favorable habitats, including temperature and aquatic bodies, taking 650 Degree/Day (DD) [38] for the *Anopheles* larvae to grow into adult mosquitoes. As a result, at an average temperature of 20 degrees per day, 32 days are required for larvae to develop into adult mosquitoes. In the meantime, there is a delay of about one month between the increase of *Anopheles* mosquitoes and the increase of temperature, and then with the increase of temperature above the allowable limit, the adult mosquitoes seek refuge in shadowy areas and vegetation and finally perish under very high temperature conditions. In this case, we were faced with two completely nonlinear trends that exhibited little correlation between them. Therefore, the most ideal evaluation for interpreting the results is the thresholding method. 

According to a study conducted by Saberi et al. [46], 522 cases of malaria were registered in Kerman Province from 2009 to 2018. A total of 88 out of 522 cases belonged to Qaleh-Ganj county. Therefore, in this study, the correlation of effective environmental factors were compared annually from 2014 to 2018 with malaria cases (Table 9). The results show that apart from the temperature factor as a definitive parameter in decision making, precipitation represented another important parameter in study areas. Precipitation increased humidity and greenery in all three study areas (due to their hot and dry climates), and provided a suitable environment for the growth of *Anopheles* larvae.

Based on the proposed method, it is possible to estimate the maximum activity of *Anopheles* mosquitoes each year without the need for extensive entomological studies. Moreover, the exact time of spraying can be determined.

## 5. Conclusions

The results of this article demonstrated that in order to accurately predict high-risk times over a yearly period for a specific area, sufficient knowledge of the climatic behavior of the area is extremely important. Moreover, employing all the effective and optimal environmental parameters simultaneously will help accurately predict the exact times of malaria outbreak for a given region. In this article, high-risk times for the three study areas of Qaleh-Ganj, Bashagard and Sarbaz were estimated based on the time series of factors affecting abundance of *Anopheles*. These times were compared with entomological data and malaria cases. Among these parameters, ALST, precipitation, soil moisture and VCI represented the most effective factors in increasing the abundance of *Anopheles*. Some factors could be removed from the final decision due to their high correlation with other parameters (e.g., TCI). It remains possible to accurately predict the appropriate times for malaria vector control operations among different areas using the proposed method and at a low cost. GEE rendered it possible to analyze the time series of climatic data in the shortest time using its processing capability and easy access to a variety of free remote sensing data. Finally, these results will help optimize the selection of data with high spatial resolution that can better locate *Anopheles* habitats for growth and survival and provide an overview of the situation in dealing with malaria outbreaks in the region. The optimization method is recommended for use in future research in order to evaluate the results and selection of the effective parameters for each region, and to perform the final analysis without the need for user supervision.

## Figures and Tables

**Table 1:** Specifications of the data used.

Data	Spatial Resolution	Temporal Resolution
Landsat8 OLI/TIRS	Visible and near infrared bands: 30 m Thermal bands: 100 m	16-Day
PERSIANN-CDR	27,830 m	Daily
MOD16A2 Terra MODIS	500 m	8-Day
NASA-USD Enhanced SMAP	10,000 m	3-Day

**Table 2:** Emissivity values based on NDVI.

NDVI	LSE
NDVI < −0.185	0.955
−0.185 ≤ NDVI < 0.157	0.985
0.157 ≤ NDVI ≤ 0.727	1.009 + 0.047 × ln(NDVI)
NDVI ≥ 0.727	0.990

**Table 3:** Adaptive thresholds to improve LST values for Qaleh-Ganj, Sarbaz and Bashagard counties, Iran.

Month	Bashagard	Qaleh-Ganj	Sarbaz
January	−9.038	−7.203	−3.269
February	−7.682	−8.139	−3.766
March	−13.109	−11.937	−7.864
April	−15.949	−17.680	−8.566
May	−16.729	−12.746	−7.599
June	−15.108	−10.506	−6.066
July	−11.403	−12.821	−10.821
August	−11.227	−12.327	−10.573
September	−11.677	−11.878	−6.560
October	−13.086	−10.136	−5.630
November	−11.782	−7.203	−3.450
December	−9.886	−5.340	−3.227

**Table 4:** Correlation between VCI and NDVI.

Study Area	Annual Interval	7-Year Interval
Bashagard	0.596727	1
Qaleh-Ganj	0.656796	1
Sarbaz	0.170149	1

**Table 5:** Correlation between TCI and LST.

Study Area	Annual Interval	7-Year Interval
Bashagard	−0.97838	−1
Qaleh-Ganj	−0.89007	−1
Sarbaz	−0.98285	−1

**Table 6:** Correlation between effective environmental factors of Bashagard County.

Correlation	ALST	NDVI	Precipitation	SSM	ET	VCI	TCI
**ALST**	1	−0.45751	−0.29909	−0.482557	−0.82527	−0.61684	−0.94131
**NDVI**	−0.45751	1	0.246871	0.231938	0.489909	0.596727	0.355025
**Precipitation**	−0.29909	0.246871	1	0.1854313	0.458859	0.301215	0.308806
**SSM**	−0.48256	0.231938	0.185431	1	0.443232	0.31193	0.562521
**ET**	−0.82527	0.489909	0.458859	0.443232	1	0.511154	0.801177
**VCI**	−0.61684	0.596727	0.301215	0.3119299	0.511154	1	0.62705
**TCI**	−0.94131	0.355025	0.308806	0.562521	0.801177	0.62705	1

**Table 7:** Correlation between effective environmental factors of Qaleh-Ganj County.

Correlation	ALST	NDVI	Precipitation	SSM	ET	VCI	TCI
**ALST**	1	−0.47511	−0.35883	−0.52238	−0.7664	−0.64931	−0.82391
**NDVI**	−0.47511	1	0.318983	0.315822	0.652536	0.656796	0.289676
**Precipitation**	−0.35883	0.318983	1	0.286829	0.510745	0.414579	0.308386
**SSM**	−0.52238	0.315822	0.286829	1	0.49336	0.481347	0.435632
**ET**	−0.7664	0.652536	0.510745	0.49336	1	0.660654	0.690367
**VCI**	−0.64931	0.656796	0.414579	0.481347	0.660654	1	0.519921
**TCI**	−0.82391	0.289676	0.308386	0.435632	0.690367	0.519921	1

**Table 8:** Correlation between effective environmental factors of Sarbaz County.

Correlation	ALST	NDVI	Precipitation	SSM	ET	VCI	TCI
**ALST**	1	−0.42695	−0.24467	−0.42271	−0.72366	−0.5002	−0.93313
**NDVI**	−0.42695	1	0.13309	0.096937	0.439853	0.171084	0.345801
**Precipitation**	−0.24467	0.13309	1	0.253735	0.239621	0.244678	0.277845
**SSM**	−0.42271	0.096937	0.253735	1	0.30915	0.310153	0.423572
**ET**	−0.72366	0.439853	0.239621	0.30915	1	0.167993	0.639373
**VCI**	−0.5002	0.171084	0.244678	0.310153	0.167993	1	0.627477
**TCI**	−0.93313	0.345801	0.277845	0.423572	0.639373	0.627477	1

**Table 9:** Correlation between effective environmental factors and malaria cases.

Average	Malaria Cases	ALST	NDVI	Precipitation	SSM	ET	VCI	TCI
2014	18	32.11317	0.088	0.396		1.285	0.183048	0.301149
2015	23	28.25653	0.082	0.395	3.42	1.003	0.196581	0.424858
2016	32	28.06708	0.078	0.622	3.32	0.823	0.137821	0.429651
2017	74	29.25537	0.088	0.767	4.01	1.071	0.268697	0.421172
2018	22	28.90742	0.077	0.181	3.22	0.673	0.134081	0.437947
**Correlation**		−0.1867	0.437	0.8153	0.9605	0.115	0.7848	0.3113

## Data Availability

Google Earth Engine. https://code.earthengine.google.com/ (accessed on 10 May 2021).

## Abbreviations

The following abbreviations are used in this article:

NDVI	Normalized Difference Vegetation Index
LST	Land Surface Temperature
ALST	Adaptive Land Surface Temperature
LSE	Land Surface Emissivity
GEE	Google Earth Engine
PERSIANN-CDR	Precipitation Estimation from Remotely Sensed Information using Artificial Neural Networks—Climate Data Record
NASA-USDA Enhanced SMAP	National Aeronautics and Space Administration-United States Department of Agriculture Enhanced Soil Moisture Active Passive
ET	EvapoTranspiration
MODIS	Moderate Resolution Imaging Spectroradiometer
TCI	Thermal Condition Index
VCI	Vegetation Condition Index
